# Oral Health Care Delivery for Children During COVID-19 Pandemic—A Retrospective Study

**DOI:** 10.3389/fpubh.2021.637351

**Published:** 2021-05-07

**Authors:** Avia Fux-Noy, Luna Mattar, Aviv Shmueli, Elinor Halperson, Diana Ram, Moti Moskovitz

**Affiliations:** Department of Pediatric Dentistry, The Hebrew University – Hadassah School of Dental Medicine, Jerusalem, Israel

**Keywords:** oral health care, dental care for children, pediatric dentistry, COVID-19 pandemic, infection control

## Abstract

**Aim:** COVID-19 outbreak and the lockdown period following was a very challenging time for pediatric dentistry. We aimed to find whether the characteristics of dental care provided to children at the Department of Pediatric Dentistry at Hadassah medical center, Jerusalem, Israel, differed between the periods, before COVID-19 outbreak, during the lockdown period and during the period that followed it.

**Materials and Methods:** We retrospectively reviewed computerized records of patients who visited the pediatric dental clinic at three different periods: pre-lockdown period, lockdown period, and post-lockdown period.

**Results:** Nine-hundred and forty-nine children were included in the study; most of them were healthy children between 3 and 6 years old. During lockdown, all scheduled appointments except for treatments under general anesthesia and deep sedation were canceled due to the government's restrictions; the frequency of treatments with non-pharmacological behavior management, general anesthesia or deep sedation was higher than in the previous or subsequent periods and the use of inhaled/conscious sedation was significantly lower. During lockdown most of the children were diagnosed with dentoalveolar abscess (32.3%), compared to 14 and 21% at the previous or subsequent periods, respectively (*P* < 0.001). Treatments combination during lockdown included more extractions, pulpectomies and pulp extirpation and less permanent restorations (*P* < 0.001). None of the staff members was infected with COVID-19 at the clinic during these periods. We concluded that dentists should be updated about Covid-19 modes of transmission and the recommended infection control measures in dental settings. Effective management protocols can help the dental staff to continue to provide efficient treatment and prevent Covid-19 contamination.

## Introduction

In early March 2020, the World Health Organization declared COVID-19, the severe acute respiratory syndrome coronavirus 2 (SARS-CoV-2) disease (1), a global pandemic (2). The virus spreads through direct transmission, such as droplet inhalation, cough, sneeze, and contact (contact with oral, nasal, and eye mucous membranes) and can also be transmitted through saliva (3). Children tend to present with similar but milder symptoms than adults (4) and their chance of being infected is smaller than adults, with the difference being more pronounced for children up to the age of ten. However, it is still undetermined whether children are more contagious than adults (5).

COVID-19 outbreak and the lockdown period following was a very challenging time for pediatric dentistry, as both dental patients and dental staff may be exposed to pathogenic microorganisms from the oral cavity and respiratory tract that are transmitted in dental settings through both direct contact (in blood, oral fluids and fluids originating in the respiratory tract) and indirect contact. Indirect contact consists of contact with contaminated objects (e.g., instruments, equipment, or environmental surfaces); contact of conjunctival, nasal, or oral mucosa with droplets (e.g., spatter) containing microorganisms and propelled a short distance (e.g., by coughing, sneezing, or talking); and inhalation of airborne microorganisms that can remain suspended in the air for long periods (6).

Members of the dental practice are exposed to tremendous risk of SARS-CoV-2 infection due to the facial proximity and the exposure to saliva, blood, and fluids originating in the respiratory tract, and the handling of sharp instruments. Furthermore, high-speed dental hand-piece without anti-retraction valves may aspirate and expel debris and fluids during dental procedures and contaminate the environment. More importantly, the microorganisms may further contaminate the air and water tubes within the dental unit (3, 6). Hence, strict adherence to principles of infection control, patient evaluation, hand hygiene and personal protective equipment is essential, as well as cleaning, disinfecting and sterilizing the equipment and the treatment environment (3). Protective measures such as disposable doctor cap and surgical mask, protective goggles, face shield, disposable isolation clothing or surgical clothes and disposable gloves, are mandatory precautions in every treatment (3).

In the absence of an international consensus on the criteria for provision of dental services, many countries restricted access or strongly discouraged non-emergency dental services during the first outbreak of the COVID-19 epidemic (7). Dental treatments with either inhaled or conscious sedation were a subject of controversy during this outbreak, as the use of nitrous oxide may increase the chance viral contamination of the gas and oxygen tubes and environmental pollution may occur when the nasal hood is not completely sealed. There is also potential risk of performing life support measures that involve handling of airways and the use of hospital resources such as intensive care units (4, 8).

On March 17th 2020 emergency lockdown was declared in Israel and the Israeli Ministry of Health issued guidelines for dealing with COVID-19 that restricted dental care to emergency treatments, limiting the use of high-speed turbine and ultrasonic scalers, and requiring thorough patient assessment and personal protective equipment to prevent the transmission of SARS-CoV-2. These guidelines remained in effect until April 27th, 2020 (9). At the following period, some restrictions on dental care were lifted, conserving the ongoing guidelines of patient evaluation, personal protective equipment, and physical distancing (10).

This retrospective study aimed to compare the characteristics of dental treatments given to children during the outbreak of COVID-19 in the Department of Pediatric Dentistry at Hadassah medical center, Jerusalem, Israel, with those given before the outbreak and those performed during the period when some restrictions were lifted.

## Materials and Methods

### Study Group and Study Design

We performed a retrospective study on computerized records of patients who visited the pediatric dental clinic in the Department of Pediatric Dentistry at Hadassah medical center at three different time periods:

1) Lockdown period- records from March 17th to April 30th, 2020. During this period, there were 25 working days (4 Sundays, 5 Mondays, 5 Tuesdays, 4 Wednesdays, 6 Thursdays).
2) Routine pre-lockdown period- records from 25 equivalent working days in the period prior to the outbreak of COVID-19. i.e., records from January 1st to February 29th, 2020.
3) Post-lockdown period- records from 25 equivalent working days in the following period when some restrictions were lifted, from May 1st to June 30th, 2020.

#### Inclusion Criteria

We included all dental treatments delivered in the clinic during the indicated periods, including treatments under general anesthesia and deep sedation.

#### Exclusion Criteria

Records with missing data, e.g., children who did not complete the dental examination were excluded.

Data collected included: age, health status, main complaint, dental diagnosis, dental care provided, behavior management technique delivered, and child's cooperation according to Frankl behavioral scale (11). For study purposes, children cooperation was further divided into negative cooperation (Frankl 1, 2) and positive cooperation (Frankl 3, 4).

### Protective Measures

Protective measures for dental staff included disposable doctor cap, disposable N95 mask, face shield, working clothes with disposable isolation clothing, and disposable latex gloves. All the equipment including the N_2_O delivery system and scavenging were sterilized between patients.

The medical center's policy is to monitor all personnel regularly for COVID-19 infection, so all member of the department of pediatric dentistry were screened regularly for COVID-19 infection from the beginning of the pandemic in Israel. During the outbreak each dental staff member was tested twice at 2-week intervals. Later, the timing of the tests was determined according to the level of morbidity in the population.

### Ethics

The study protocol was approved by the Institutional Human Subjects Ethics Committee of Hadassah Medical Organization IRB, Jerusalem, Israel (IRB 0385-20-HMO).

### Statistical Analysis

Data on each appointment at the three periods were recorded. Chi-Square Tests were used to compare patients' characteristics (age, health status, visit purpose\main complaint, dental diagnosis, dental care provided, behavior management technique adopted, and child's cooperation) between the three periods. The software used for statistical analysis was SPSS 20.0 software for Windows (IBM Corp., Armonk, NY, USA). All tests applied were two tailed, and a *p*-value of 0.05 or less was considered a statistically significant.

## Results

**Figure 1:**
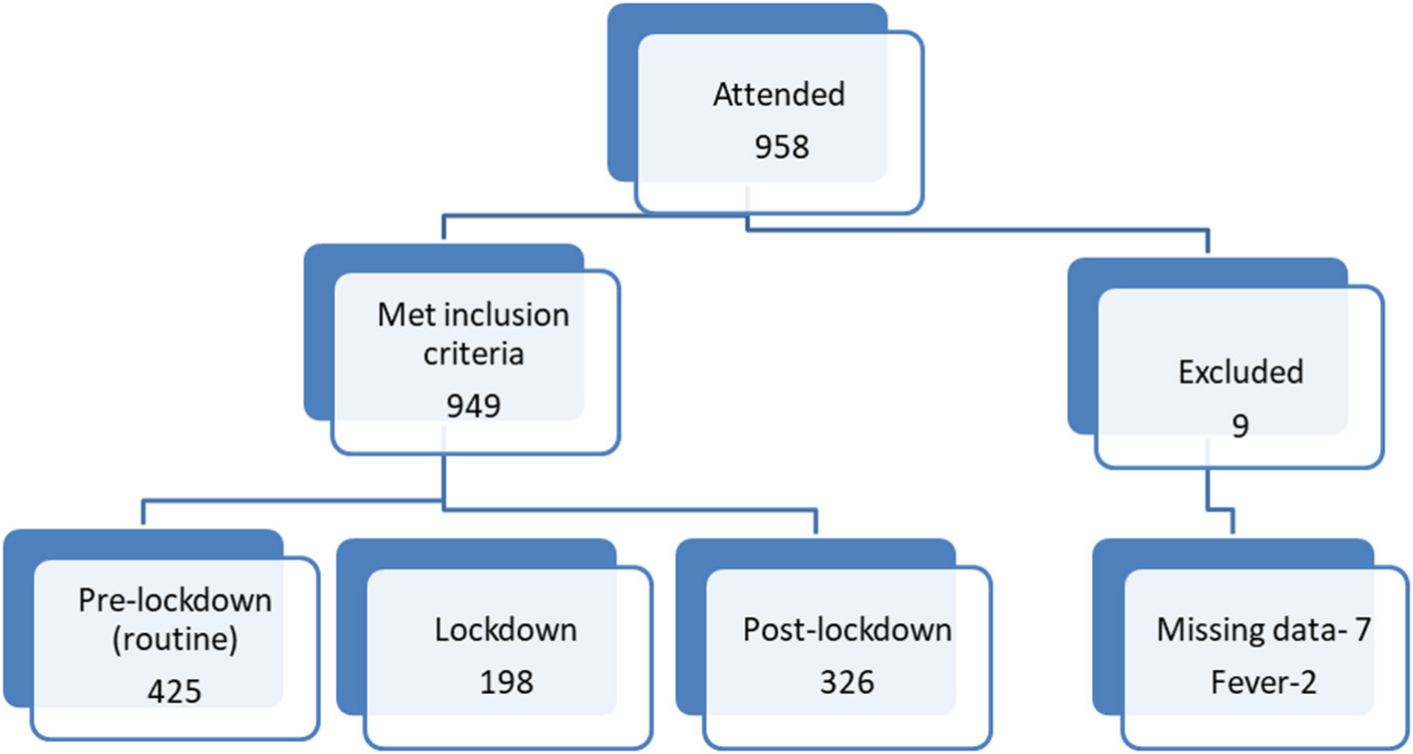
Patient allocation flow diagram.

A total of 958 children attended the clinic at the relevant periods. Nine were excluded from the study, all of them from lockdown period group; seven due to missing data in the records, two had high fever on arriving and were referred to the Department of Emergency Medicine. Nine-hundred and forty-nine children were included in the study (Figure 1): 425 children in routine pre-lockdown group, 198 children in the lockdown period group (53.4% decrease in visits compared to pre-lockdown), and 326 children in post lockdown period group (23.3% decrease in visits compared to pre-lockdown). The age range of the total study group was 6 months to 15 years, mean age was 5.49 years, median age was 5 years. There was no difference in age distribution between groups. Most of the children were healthy. Patients' characteristics are presented in Table 1.

**Table 1:** Patients' characteristics.

		**Pre-lockdown**	**Lockdown**	**Post lockdown**	***p*-value**
	**Total number of attending children**	**425 (100%)**	**198 (100%)**	**326 (100%)**	
Visit purpose\main complaint	Dental emergency- pain	84 (19.8%)	99 (50%)	81 (24.8%)	0.001
	Dental emergency- swelling\post antibiotic course	17 (4%)	26 (13.1%)	15 (4.6%)	
	Dental emergency- Dental trauma\trauma follow up	29 (6.8%)	29 (14.6%)	40 (12.3%)	
	Caries, dental examination	85 (20%)	10 (5.1%)	48 (14.7%)	
	Other complaints	12 (2.8%)	12 (6.1%)	9 (2.8)	
	Scheduled dental treatment without sedation	17 (4%)	0	9 (2.7%)	
	Scheduled dental treatment under inhaled/conscious sedation	155 (36.4%)	0	100 (30.7%)	
	Scheduled dental treatment under deep sedation	6 (1.4%)	5 (2.52%)	4 (1.22%)	
	Scheduled dental treatment under general anesthesia	20 (4.7%)	17 (8.58%)	20 (6.13%)	
Age (years)	Range	1–15	0.5–12	0.9–15	
	Mean	5.76	5.46	5.16	
	Median	5	5	4	
	0–3	47 (11.1%)	22 (11.1%)	39 (12%)	0.125
	>3–6	183 (43.1%)	92 (46.5%)	173 (53.1%)	
	>6–9	113 (26.6%)	50 (25.3%)	71 (21.8%)	
	>9	82 (19.3%)	34 (17.2%)	43 (13.2%)	
Health status	Healthy	355 (85.5%)	178 (89.9%)	281 (86.2%)	0.102
	Medical problems	70 (16.5%)	20 (10.1%)	45 (13.8%)	
	**Patients who seek emergency treatment**	**277 (100%)**	**176 (100%)**	**193 (100%)**	
Dental diagnosis	Dento-alveolar abscess, fistula	31 (14.7%)	60 (32.3%)	38 (21%)	0.001
	Reversible\irreversible pulpitis	29 (13.7%)	39 (21%)	21 (11.6%)	
	Caries	99 (46.9%)	49 (26.3%)	65 (35.9%)	
	Dental trauma	17 (8.1%)	22 (11.8%)	30 (16.6%)	
	Others	22 (10.4%)	15 (8.1%)	17 (9.4%)	
	**Patients treated operatively**	**242 (100%)**	**122 (100%)**	**186 (100%)**	
Behavior management	Non-pharmacological BM	23 (9.5%)	27 (22.1%)	12 (6.5%)	0.001
	Inhalation sedation (nitrous oxide)	94 (38.8%)	32 (26.2%)	57 (30.6%)	
	Conscious sedation	99 (40.9%)	41 (33.6%)	93 (50%)	
	Deep sedation	6 (1.4%)	5 (2.52%)	4 (1.22%)	
	General anesthesia	20 (4.7%)	17 (8.58%)	20 (6.13%)	
	**Patients with data on cooperation**	**371 (100%)**	**132 (100%)**	**290 (100%)**	
Cooperation	Negative	95 (25.6%)	31 (23.5%)	92 (31.7%)	0.115
	Positive	276 (74.4%)	101 (76.5%)	198 (68.5%)	

*The bold values represents the size of the study population of the mentioned characteristic*.

### Purpose of Visit

At lockdown period, all scheduled appointments (except for treatments under general anesthesia and deep sedation) were canceled according to government's restrictions (Table 1). This led to a significant alteration in the visit purpose\main complaints (*P* < 0.001), as during the lockdown period most patients arrived with an emergency such as dental pain, swelling, or dental trauma. Of note, even when some of the restrictions were lifted, the amount of scheduled treatments with/without inhaled or conscious sedation did not return to its pre-pandemic volume because of the need to perform more stages in the disinfecting procedures between patients.

### Behavior Management Techniques

The frequency of treatments with non-pharmacological behavior management, general anesthesia or deep sedation was higher during lockdown period than in the previous or subsequent periods. Despite the increase in the amount of treatments with a non-pharmacological method, during lockdown over 50% of the patients were treated with nitrous oxide (either exclusively or combined with oral sedative), while post lockdown over 80% of patients were treated with nitrous oxide (*P* < 0.001), as listed in Table 1. No significant difference was found in children's cooperation between the three time periods (*p* = 0.115, see Table 1).

### Dental Findings

In accordance with the main complaints, during lockdown more children were diagnosed with dentoalveolar abscess (32.3%), compared with 14 and 21% at the preceding and following periods, respectively (*P* < 0.001). Caries was the most prevalent finding at the periods prior to and following lockdown (47 and 36%, respectively, *P* < 0.001). Distributions of oral and dental findings among children attending emergency appointment are presented in Table 1.

### Dental Treatments Provided

During lockdown, 75 (37.9%) of the children required at least one extraction, compared to 75 (17.6%) at the pre and 72 (22.1%) at post lockdown periods (*P* < 0.001). Extractions and pulpectomy were performed more frequently at lockdown compared to the other periods, while other treatments such as permanent restorations (such as composite resin restorations and stainless-steel crowns) were performed less frequently. These differences were statistically significant, as shown in Table 2.

**Table 2:** Treatments provided according to periods (including dental emergencies and scheduled appointments).

		**Pre-lockdown**	**Lockdown**	**Post lockdown**	***p*-value**
		***n* = 425 (100%)**	***n* = 198 (100%)**	***n* = 326 (100%)**	
Type of treatment (at least one tooth per patient)	Examination/referral to another department	178 (41.9%)	65 (32.8%)	136 (41.7%)	0.072
	Extraction	75 (17.6%)	75 (27.9%)	72 (22.1%)	0.001
	Pulpectomy, pulp extirpation	33 (7.8%)	33 (16.7%)	36 (11%)	0.004
	Pulpotomy, partial pulpotomy	47 (11.1%)	22 (11.1%)	27 (8.3%)	0.339
	Permanent restoration	197 (46.4%)	38 (19.2%)	114 (35%)	0.001
	Others	15 (3.5%)	15 (7.6%)	13 (4%)	0.066

None of the staff members has been diagnosed with COVID-19 during the three periods except for one dental assistant who had been infected with the virus on a social occasion and was absent from the department until full recovery after 2 weeks.

## Discussion

Caries prevention represents the gold standard toward which health professionals specialized in pediatric dentistry should always be oriented. This is even truer in times of health emergency.

We found a decrease in the number of patients looking for emergency dental care during lockdown period compared with routine pre-pandemic period and, to a lesser extent, also post lockdown.

The pandemic has affected not only dental but all medical emergency seeking patients, as shown by Wong et al. (12). There is increasing evidence that patients with medical emergencies are avoiding the emergency departments for fear of contracting COVID-19, leading to increased morbidity and mortality (12). Avoidance of routine dental treatment may lead to deterioration in oral health and increase in dental infections, caries, neglect of dental trauma and periodontal disease. Similar to our findings, Guo et al. reported 38% decrease in the number of patients visiting the dental emergency clinic in Beijing, China at the beginning of the COVID-19 pandemic (13). Tobias et al. reported a decrease in dental emergency treatment in the period between March 17th to April 30th, 2020 in a public health system in Israel (14).

The fact that in our clinics none of the staff members and the patients was infected with COVID-19 during the three periods studied is encouraging. It suggests that it may be safe to continue regular treatments under strict infection control regulations, when personal protective measures are put in place and adhered to, and thus avoid dental deterioration in children in need of treatments.

In the present study, the decrease in the patients' numbers at the post lockdown period reflects the continuing restrictions set by the Health Ministry. The need to reduce crowding in the waiting room and to sterilize all surfaces and equipment between patients requires longer appointment for each patient, thus leading to a decrease in the number of treatments that can be provided. However, the number of patients treated under general anesthesia or deep sedation stayed approximately constant during the three periods because such treatments were allowed within the limits of restrictions.

Behavior management is especially important in limiting infection. Restless, crying children spread more aerosol compared to calm children, so appropriate and skillful behavior management is the first and most important basic technique to minimize the probability of SARS-CoV-2 cross-infection. Dental care to children involves various methods for behavior management, including inhaled or conscious sedation, which were a subject of controversy during COVID-19 outbreak; for example, Simpson et al. (15) avoided inhalation sedation due to difficulties disinfecting the reusable nasal hoods and tubing. This led them to use non-pharmacologic behavior management techniques which they reported effective in over a third of patients who needed extraction and trauma management under local anesthesia (15). We have used a higher rate of non-pharmacologic behavior management during lockdown compared with the other periods, as less patients were treated with nitrous oxide (either exclusively or combined with oral sedative) at lockdown. The fact that no case of cross-infection of patients or staff was reported at our department suggests that the use the nitrous oxide is safe if managed and sterilized as instructed by the manufactures.

Some treatments were performed more frequently during lockdown compared with other periods, such as extractions and pulpectomies, while other treatments were performed less frequently, such as permanent restorations. Simpson et al. also reported that irreversible pulpitis and dental trauma were the most common reasons children accessed the service, largely (49%) resulting in extractions (15). The restrictions on dental treatment that could be provided during lockdown and uncertainty about the duration of the restrictions, may have led the dentists to more radical treatments such as extractions. Another possible reason is that the increase in extractions reflects the severity of the dentals condition that led the patients to seek help.

As seen by our findings, the need to avoid aerosol- producing procedures results in a decrease in the performance of permanent restorations. So far, no study has evaluated disease transmission via aerosols in dental settings and there has been no evidence indicating viral contamination through aerosols. Furthermore, it was impossible to conclude whether interventions that aim to reduce aerosol production during dental procedures prevent the transmission of infectious diseases (16).

Al-Halabi et al. (17) suggested that minimally invasive treatments be used for caries management to minimize aerosol generating procedures that may result in viral cross-infection. Treatment alternatives for asymptomatic teeth include sealing non- cavitated caries, using fluoride varnish and resin infiltration to arrest non-cavitated caries, atraumatic or alternative restorative technique (ART), interim therapeutic restorations (ITR), indirect pulp capping (IPC), the Hall technique (HT), and the use of Silver Diamine Fluoride (SDF). We suggest that further research is needed to establish if ART presents as an effective alternative to conventional methods in the post-COVID-19 era. We suggest that effective management protocols can enable the dental staff to safely provide efficient treatments and prevent COVID-19 contamination.

## Data Availability Statement

The data analyzed in this study is subject to the following licenses/restrictions: The data analyzed are part of medical records of patients. Requests to access these datasets should be directed to fuxavia@gmail.com.

## Ethics Statement

The studies involving human participants were reviewed and approved by Institutional Human Subjects Ethics Committee of Hadassah Medical Organization IRB, Jerusalem, Israel (IRB 0385-20-HMO). Written informed consent from the participants' legal guardian/next of kin was not required to participate in this study in accordance with the national legislation and the institutional requirements.

## Author Contributions

AF-N, MM, and DR conceived the ideas and designed the work. LM collected the data. AF-N, MM, and LM analyzed the data. AF-N, AS, EH, DR, and MM led the writing. All authors contributed to the article and approved the submitted version.

## Conflict of Interest

The authors declare that the research was conducted in the absence of any commercial or financial relationships that could be construed as a potential conflict of interest.
